# Effectiveness of Epidural Steroid Injection Depending on Discoradicular Contact: A Prospective Randomized Trial

**DOI:** 10.3390/ijerph20043672

**Published:** 2023-02-19

**Authors:** Dino Budrovac, Ivan Radoš, Dijana Hnatešen, Ivana Haršanji-Drenjančević, Ozana Katarina Tot, Franjo Katić, Iva Lukić, Sonja Škiljić, Nenad Nešković, Iva Dimitrijević

**Affiliations:** 1 Faculty of Medicine Osijek, Josip Juraj Strossmayer University of Osijek, 31000 Osijek, Croatia; 2 Department of Anaesthesiology, Reanimatology and Intensive Care, University Hospital Centre Osijek, 31000 Osijek, Croatia; 3 Nursing Institute “Professor Radivoje Radić”, Faculty of Dental Medicine and Health Osijek, Josip Juraj Strossmayer University of Osijek, 31000 Osijek, Croatia; 4 Clinical Department of Diagnostic and Interventional Radiology, University Hospital Centre Osijek, 31000 Osijek, Croatia; 5 Clinical Institute for Laboratory Diagnostics, University Hospital Centre Osijek, 31000 Osijek, Croatia

**Keywords:** herniated disc, transforaminal epidural steroid injection, low back pain, disability, radiculopathy

## Abstract

Lumbar radicular pain is a major public health and economic problem. It is among the most common reasons for professional disability. The most common cause of lumbar radicular pain is intervertebral disc herniation, which results from degenerative disc changes. The dominant pain mechanisms are direct pressure of the hernia on the nerve root and the local inflammatory process triggered by intervertebral disc herniation. Treatment of lumbar radicular pain includes conservative, minimally invasive, and surgical treatment. The number of minimally invasive procedures is constantly increasing, and among these methods is epidural administration of steroids and local anesthetic through a transforaminal approach (ESI TF). The aim of this research was to examine the effectiveness of ESI TF as measured by a visual analog scale (VAS) and the Oswestry Disability Index (ODI), depending on whether there is contact between the herniated intervertebral disc and the nerve root. In both groups of participants, there was a significant reduction in pain intensity, but there was no significant difference between the groups. In the group with disc herniation and nerve root contact, the only significant reduction was in pain intensity (*p* < 0.001). There were no significant differences in measurements in other domains of the ODI. In the group without disc herniation and nerve contact, there was a significant difference in all domains except weight lifting. In the group without contact, there was significant improvement after 1 month (*p* = 0.001) and 3 months (*p* < 0.001) according to the ODI, while there was no significant improvement in the group with contact. In addition, there were no significant differences in the distribution of participants based on the ODI and whether disc herniation and nerve contact was present. The results suggest that transforaminal epidural administration of steroids is a clinically effective method for treating lumbar radicular pain caused by intervertebral disc herniation in people with and without nerve root contact, without significant differences.

## 1. Introduction

Lumbar radicular pain is a major public health problem. In the medical literature, it is also called sciatica, defined as pain that spreads along the sciatic nerve to the lower extremities. In addition to pain, muscle weakness and leg numbness may also be present, significantly reducing the functionality and quality of life of patients [1,2]. It is the significant cause of disability worldwide [3]. The prevalence of lumbar radicular pain is 12.2–43% [4]. Herniated intervertebral discs cause approximately 85% of cases of lumbar radicular pain [5].

Degenerative changes that cause disc protrusion are the most common cause of lumbar radicular pain [6]. In addition to disc protrusion, spinal canal stenosis, foraminal stenosis, and stenosis caused by cysts, tumors, or extraspinal pathology can also cause lumbar radicular pain [7]. The two main pathophysiological mechanisms are the local inflammatory process and the pressure of the herniated intervertebral disc on the nerve root [8]. The diagnosis is made based on the patient’s history and a clinical examination [9]. It is necessary to exclude other pathological states such as cancer, trauma, infection, and cauda equina syndrome, which is characterized by urinary retention and disorder of the tone of the anal sphincter. If the symptoms last longer than 12 weeks or there is worsening of pain or the development of a neurological deficit, an MRI of the lumbar spine is indicated [1]. Lumbar radicular pain is usually self-limiting; within a few weeks, the pain decreases with or without treatment [10]. The first line of treatment is a conservative approach [11], which could be partially compromised if pharmacotherapy is avoided due to the patient’s impaired cognitive or emotional status, which have been identified as potential predictors of treatment success [12]. If conservative treatment has not achieved the desired outcome, epidural administration of local anesthetic and steroids is considered [11]. Although this treatment approach is generally accepted around the world and the number of such procedures is increasing, especially using the ESI TF approach, the guidelines of the Danish Health Authority do not recommend this modality for lumbar and lumbar radicular pain in the first 12 weeks from the onset of symptoms [13]. 

ESI is effective for short-term treatment of pain and postponing surgery, but the evidence for a longer-term effect is lacking [14]. It is generally known how the application of corticosteroids achieves the analgesic effect [15].

The most common approaches to the epidural space are interlaminar (ESI IL), caudal, and transforaminal. Both ESI TF and ESI IL lead to a significant reduction in pain and disability; there is no significant difference between the two, although research has shown that the transforaminal approach is more effective and achieves an equivalent or better result with the use of a lower dose of corticosteroids [16,17]. ESI TF is safe, but severe neurological complications, such as cord infarct, have also been described [18]. The antinociceptive properties of local anesthetics and steroids are known. The “washing-out” effect reduces local inflammatory factors, and steroids have anti-inflammatory properties [19]. In some patients, the effect of ESI lasts for several months and enables them to perform physical therapy and reduce the use of analgesics, while in other patients, the effect is short-lived, only a few days, or there is no effect [20]. If the pain persists despite attempting all modalities of conservative and minimally invasive treatment, then the method of choice is microdiscectomy, which is the gold standard of treatment [21]. To date, research has been conducted in which the characteristics of the herniated disc and the relationship of the hernia to the nerve root and the impact on reducing pain and disability were investigated, but the results were often contradictory [14,22,23,24].

Therefore, the aim of this study was to examine the effectiveness of ESI TF in the treatment of lumbar radicular pain caused by herniated intervertebral discs depending on the contact between the herniated disc and the nerve, as measured by the VAS and the ODI.

## 2. Materials and Methods

### 2.1. Study Design

**Figure 1:**
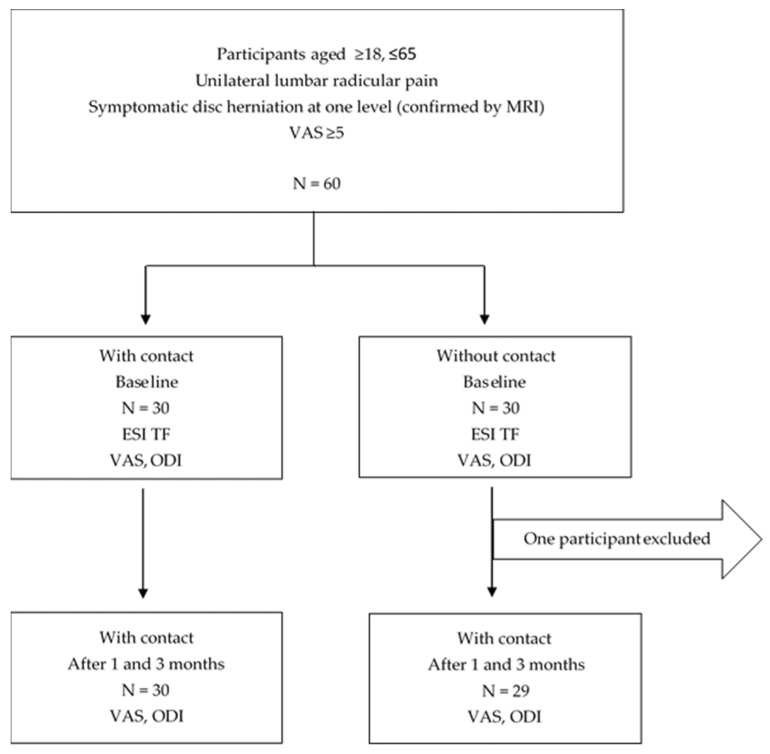
Study design.

This prospective randomized trial was conducted between January 2020 and November 2022 at the Clinical Department of Pain Management at the University Hospital Osijek after obtaining the approval of the Ethics Committee of Osijek Clinical Hospital Center (R2-18577/2019) and in accordance with the Declaration of Helsinki. All participants received a written consent form describing the procedure to be performed, and the procedure was explained to patients orally. After signing the informed consent, the patients were divided into 2 groups based on whether there was discoradicular contact. This study included 60 participants, among which 1 participant dropped out, so the statistical analysis was made on 59 participants. At the beginning of this study, before ESI TF (baseline), and 1 and 3 months after ESI TF, the participants rated their pain intensity by the VAS and their degree of disability was measured by the ODI (Figure 1).

### 2.2. Participants

The included participants were between the ages of 18 and 65 years with unilateral lumbar radicular pain, with symptomatic disc herniation at one level verified by magnetic resonance imaging, and pain intensity measured on the VAS from 0 to 10 or ≥5. Exclusion criteria were as follows: age < 18 or >65 years; refusal to participate in this study; central stenosis of the lumbar canal; lumbar radicular pain due to causes other than intervertebral disc herniation; pregnancy; allergy to steroids, local anesthetics, or contrast media; positive history of prolonged bleeding; local or systemic infection; and previous lumbar spine surgery at the currently observed level.

### 2.3. ESI TF Treatment Procedure

ESI TF was performed in the operating theater according to the rules of asepsis. Each participant was placed in the prone position on the operating table. Before and during ESI TF, the participant’s vital parameters were monitored. The skin at the planned needle insertion site was aseptically prepared and draped. ESI TF was performed under the control of a fluoroscope using an oblique projection of 20–30° and a lateral projection. Nonionic contrast was used to confirm the needle tip’s position in the epidural space and to exclude intrathecal and intravascular positions. Then, a solution of 5 mL of 0.25% levobupivacaine and 40 mg of methylprednisolone was injected. After the procedure, the participants were monitored and discharged home with stable vital parameters. All participants were outpatients.

### 2.4. Outcome Measure

#### 2.4.1. The Visual Analog Scale (VAS)

A visual analog scale (VAS) was used to measure pain intensity. The scale consists of a solid line divided by numbers from 0 to 10, with 0 on the far left, which indicates the absence of pain, and 10 on the far right, which indicates unbearable pain. The VAS is the most commonly used measure of pain in clinical practice, with a high degree of resolution. The patient marks on the line the point that they feel represents their perception of their current state [25].

#### 2.4.2. The Oswestry Disability Index (ODI)

The ODI questionnaire was first published in 1980 and underwent several subsequent revisions [26,27]. The most recent version is the ODI 2.1b, and the author recommends that for any new study, the original version 2.1b must be used. Regardless, for now, translations of the ODI 2.1a can be used [28]. The Croatian version of the ODI 2.1a was used due to the available validation in the Croatian language. The Croatian version of the ODI has acceptable psychometric properties [29].

The ODI 2.1a consists of 10 sections (pain intensity, personal care, lifting, walking, sitting, standing, sleeping, sex life, social life, and traveling), each containing 6 statements about related activities. The responses are scored on a 0–5 scale. The total score is expressed as a percentage of the maximum score, with lower scores indicating lower levels of disability and vice versa. Cut-off values discriminate between minimal (≤20% of total score), moderate (21–40% of total score), and severe (41–60% of total score) disability, as well as crippled (61–80% of total score) and bedbound (81–100% of total score) patients [30].

### 2.5. Statistical Methods

Categorical data were represented by absolute and relative frequencies. Differences in categorical data between independent groups were tested with the 2-tailed test and, if necessary, with Fisher’s exact test. Differences between measurements were tested with the McNemar–Bowker test or the marginal homogeneity test. The normality of the distribution of numerical variables was tested with the Shapiro–Wilk test. The median and the limits of the interquartile range describe continuous data. To test the differences of continuous variables between two independent groups (comparison between groups with and without contact), the Mann–Whitney U test was used, and between measurements, the Friedman test (post hoc Conover test). Spearman’s correlation coefficient was used to express the assessment of connection. All *p*-values are two sided. The significance level was set at α < 0.05. MedCalc^®^ Statistical Software version 20.123 (2022; MedCalc Software Ltd., Ostend, Belgium; https://www.medcalc.org) and IBM SPSS 23 (2015; IBM Corp., Armonk, NY, USA) were used for data analysis.

## 3. Results

This study was conducted on 59 participants, among which 30 (51%) had contact and 29 (49%) had no contact between disc herniation and nerve. The median age of participants was 49 years (interquartile range, 41 to 58 years), and the age range was 26 to 65 years.

Most of the participants (N = 45, 76%) had L5/S1 involvement. Among the participants, 44 (75%) had a secondary education, and 22 (37%) were employed (Table 1.). 

There were no significant differences in pain intensity measured at three measurement points between participants with and without disc herniation and nerve contact. Before ESI TF, the pain was significantly more severe in comparison to other measurements in both the group with contact (Friedman’s test, *p* < 0.001) and the group without contact (Friedman’s test, *p* < 0.001). Among all participants, pain values were significantly higher before than after ESI TF (Friedman’s test, *p* < 0.001) (Table 2).

Improvement in pain intensity by 50% or reduction in VAS score was distributed into three ratings. There were no significant differences in pain improvement between groups according to the VAS scale and measurement points (Table 3).

Among all participants, there was a significant improvement in all domains and the overall scale, except for lifting weights and social life (Table 4).

Spearman’s correlation coefficient was used to evaluate the relationship between age and the total ODI before and 1 and 3 months after ESI TF. In the group of participants who had contact between a herniated disc and a nerve before ESI TF, older participants had more pronounced difficulties in the domain of travel (Rho = 0.544). Older participants had difficulty sleeping 1 month after ESI TF (Rho = 0.502), and the whole scale is worse (Rho = 0.401). Three months after the procedure, older participants had problems with walking (Rho = 0.444), sleeping (Rho = 0.650), and social life (Rho = 0.449), and the entire ODI was worse (Rho = 0.491). Among participants without contact between the herniated disc and the nerve, only 3 months after ESI TF, older participants expressed difficulty lifting loads (Rho = 406).

Before ESI TF, difficulty standing (Mann–Whitney U test, *p* = 0.04) and traveling (Mann–Whitney U test, *p* = 0.03) were significantly more pronounced in participants without disc herniation and nerve contact. In contrast, for other measurement points, there were no significant differences between the groups. In the group of patients with disc herniation and nerve contact, the only significant reduction was in pain intensity (Friedman’s test, *p* < 0.001). In other domains, there were no significant differences in measurements. In the group without disc herniation and nerve contact, there were significant differences in all domains, except the load-lifting domain, in which no significant change was recorded for the three measurement points (Table 5).

There were no significant differences in the distribution of participants according to the ODI or the presence of disc herniation and nerve contact. There was no significant improvement according to the ODI in the group with contact (Table 6). There was significant improvement after 1 month (test of marginal homogeneity, *p* = 0.001) and 3 months (test of marginal homogeneity, *p* < 0.001) according to the ODI before the procedure in the group without contact between the disc herniation with the nerve (Table 7). 

## 4. Discussion

The aim of this study was to examine the effectiveness of ESI TF in patients with lumbar radicular pain caused by intervertebral disc herniation, depending on the presence of discoradicular contact or not. Pain intensity was measured by the VAS, and the degree of disability was measured by the ODI.

In this study, the median age of participants was 49 years, most participants had a secondary education (75%), and a large proportion were on sick leave (34%). These results align with other research confirming that lumbar radicular pain most often affects the working population and represents a significant economic problem due to work disability and sick leave [22]. The most frequently affected segment was L5/S1 (76%), which corresponds to other study results [22,23,31,32]. The effectiveness of transforaminal epidural administration of steroids and local anesthetic is unquestionable [33,34]. A meta-analysis by Helm et al. determined that there is level 1 evidence supporting the use of epidural corticosteroids and local anesthetic to treat radicular pain caused by disc herniation. The effectiveness was determined by the measurement of reduced pain and improved functional status after 3 and 6 months [18].

MRI findings do not always correlate with the clinical picture or treatment outcome after ESI, thus there is a need to identify factors so that patients and doctors can know what treatment outcome they can expect. Numerous factors have been studied as possible predictors of treatment outcomes. Serum markers and MRI findings have been studied the most often [35]. The previous research indicates no clear position regarding the predictive value of MRI findings [23,35]. In this study, the focus was on MRI, and the treatment outcome was observed depending on the presence of contact between the herniated intervertebral disc and the nerve root before ESI TF. In addition, we considered minimal clinically significant improvement to be a reduction in pain intensity measured by the VAS by 3 or at least 50% of the initial values [36]. We examined the effectiveness of ESI TF within each group as measured by the VAS and the ODI. A reduction in pain intensity by 50% or more or by a minimum of 3 points among those with herniated disc and nerve contact after 1 month was reported by 67% of participants, and after 3 months by 60% of participants. A reduction in pain by 50% or more or by a minimum of 3 points among those without disc and nerve contact after 1 month was reported by 70% of participants, and after 3 months by 68% of participants. The reduction in pain intensity was significant in both the group with contact and the group without contact, but there was no significant difference between the two groups at any measurement point.

The results obtained in this study align with the research by Tecer et al., who examined MR findings as predictors of treatment outcomes. In addition to the contact itself, the type of hernia (bulging, protruding, extruding), hernia location (central, subarticular, foraminal, extraforaminal), high-intensity zone (HIZ), and nerve root impingement (NRI) were observed. The median age of the participants was similar to that in our study (45.3 ± 12.5 years), as well as the most affected segment (L5/S1). Among all participants, there was a statistically significant reduction in pain intensity by at least 50% in 42% of participants after 2 weeks and in 56% of participants after 3 months. During the follow-up period, there were no significant differences in reduced pain intensity depending on the type and location of the hernia. In participants with nerve root compression, the success rate was higher but not statistically significant [22]. The reduction in pain intensity after 3 months was similar to this study, which was 68% of all participants regardless of whether there was contact between the herniated disc and any nerves. The results differ depending on the compression of the nerve root. Specifically, in this study, the effectiveness was lower in participants with contact than in those without contact even after 1 month (67% vs. 72%), and after 3 months (60% vs. 76%), but the difference is not statistically significant.

In a Korean study, there was no significant difference between responders and nonresponders based on the type and size of the hernia. The location of the hernia and the degree of nerve root compression proved to be good predictors of treatment outcome. Specifically, participants with central and extraforaminal hernias had better treatment outcomes, while those with a subarticular hernia had worse treatment outcomes. Those with a lower degree of compression had a more painful treatment outcome than those with a higher degree of compression [23]. The results of the previous study on effectiveness depending on the presence of contact of the hernia with the nerve root correspond to the results of our study. In a study by Lee et al., the results showed that other factors such as high T2 signal, relationship to the nerve root, reduction in disc height, degree of degenerative disc changes, and osteophytes did not prove to be statistically significant [31]. A group of Australian authors examined the radiological characteristics of discs that could serve as predictors of treatment outcomes. Their results showed that patients with low-grade compression improved in 75% of cases, while patients with high-grade compression improved in 26% of cases.

In patients with a lower degree of compression, it is considered that the main pathophysiological mechanism of pain is the local inflammatory process, which is why ESI is effective. It is less effective in patients with a higher degree of compression, but even then the inflammatory component should not be ignored. The effectiveness of ESI TF, defined as a reduction in pain by at least 50% in all participants, was found in 53.5% of cases. No correlation was observed between efficacy and location and type of hernia [24]. It is known that radiculopathy is caused by a local inflammatory process and not only by mechanical compression, which could explain why pain intensity is not always correlated with contact between the disc herniation and the nerve [37,38]. Therefore, as they may be predictors of better or worse treatment outcomes, the determination of serum markers should be included, not only MR findings.

In a study by Kaufmann et al., the results showed that the effectiveness of ESI TF after 2 weeks was 40.9% and after 2 months was 45.6%, as measured by the Roland–Morris Disability Questionnaire [39]. In research conducted by Viton et al., it was established that pain reduction after ESI TF to treat lumbar radicular pain was more significant in patients up to 50 years of age compared to older patients, whereas in our study, there was no significant difference regarding age [40]. In Lee et al.’s study, the results showed that those in the group with better treatment outcomes were older than those with worse treatment outcomes [31]. In the group of participants with contact before ESI TF (baseline) and after 1 and 3 months, their ODI was 61%, 54%, and 53%, and in the group without contact, their ODI was 70%, 50%, and 54%, respectively. The initial functional capacity was comparable between the two groups (61% vs. 70%), and the median ODI for all participants, with or without contact, was 64%. The group with disc herniation and nerve contact experienced a significant reduction in pain intensity, while there were no significant differences in measurements in other domains. In the group without disc herniation and nerve contact, functionality was significantly improved in all domains except load-lifting, for which no significant change was recorded for the three measurement points. There was greater initial disability in participants without contact compared to those with contact (70% vs. 64%), although it was not significant and did not correlate with higher pain intensity in patients with contact compared to those without contact (VAS 8 vs. VAS 7). There was significant improvement after 1 and 3 months according to the ODI before ESI TF in the group without contact, while in the group with contact there was no significant improvement according to the ODI. There were no significant differences in the distribution of participants according to the ODI and those with and without disc herniation and nerve contact.

In a study by Rados et al., in which the patients were not divided into groups according to whether there was contact of the disc herniation and the nerve root, the initial disability was 53%, while it was 38% after 6 months [16]. The degree of disability was lower than in this study, which could be explained by the lower initial VAS score, which was 6.7 in that study, while in this study, the median VAS was 8. Kaufmann et al. examined the effectiveness of ESI TF as measured by the Roland–Morris Disability Questionnaire. After 2 weeks, 31.9% of patients responded, and after 2 months 41.3% responded. The reduction in disability was significant [39]. Moreover, looking at disability due to low back pain, the study results obtained with the SF-36 questionnaire, which includes the domain of physical functioning, indicate that participants scored the lowest in the area of physical functioning. They stated that their difficulty with physical functioning led them to reduce the time they spent working or caused such discomfort that they could not perform their planned activities [41].

The literature reports varying rates of effectiveness of ESI TF. It is not equally effective in all patients, so there is a need to identify clinical, radiological, and other predictors to determine which patients will benefit.

## 5. Limitations

This study included only participants from one clinical hospital center, which may affect the accuracy of the interpretation of the results. It is important to be aware that the questionnaires have biases and patients’ scores are subjective when assessing pain intensity. Although this study was prospective and randomized, and the required number of participants was obtained by statistical calculation, it would be desirable to conduct a study with larger numbers of participants and observed variables. In addition, the drugs used for ESI TF are not uniform in the literature, which could also affect the obtained results.

## 6. Conclusions

Transforaminal epidural administration of steroids is a clinically effective method of treating lumbar radicular pain caused by intervertebral disc herniation in patients with or without disc herniation and nerve root contact, without significant differences.

## Figures and Tables

**Table 1:** Basic characteristics of participants.

	Number (%) of Participants			*p* *
	With Herniated Disc and Nerve Contact	Without Herniated Disc and Nerve Contact	Total	
Gender				
Male	10 (33)	9 (31)	19 (32)	0.85 ^†^
Female	20 (67)	20 (69)	40 (68)	
Age groups				
Up to 50 years	12 (40)	18 (62)	30 (51)	0.09
50 and over	18 (60)	11 (38)	29 (49)	
Level				
L4/L5	2 (7)	7 (24)	9 (15)	0.10
L5/S1	24 (80)	21 (72)	45 (76)	
L3/L4	4 (13)	1 (3)	5 (8)	
Education				
Primary school	4 (13)	4 (14)	8 (14)	0.75
High school	23 (77)	21 (72)	44 (75)	
Bachelor’s degree	1 (3)	0	1 (2)	
Master’s degree	2 (7)	4 (14)	6 (10)	
Working status				
Employed	11 (37)	11 (38)	22 (37)	0.78
Unemployed	3 (10)	4 (14)	7 (12)	
Retired	4 (13)	2 (7)	6 (10)	
On sick leave	9 (30)	11 (38)	20 (34)	
Invalid	3 (10)	1 (3)	4 (7)	

* Fisher’s exact test; ^†^ χ^2^ test.

**Table 2:** Evaluation of the VAS at three measurement points.

VAS	Median (Interquartile Range)						*p* ^†^
	Baseline	*p* *	One Month from ESI TF	*p* *	Three Months from ESI TF	*p* *	
With herniated disc and nerve contact	8 (7–8)	0.65	4 (3–6)	0.43	5 (3–7)	0.20	<0.001 ^‡^
Without herniated disc and nerve contact	7 (7–9)		4 (0–6)		4 (1–6)		<0.001 ^‡^
All participants	8 (7–9)		4 (1–6)		5 (2–6)		<0.001

* Mann–Whitney U test (comparison between groups with and without contact); ^†^ Friedman test (post hoc Conover test). ^‡^ At the *p* < 0.05 level, values before ESI TF (baseline) were significantly higher than all other measurements.

**Table 3:** The reduction in pain intensity measured by the VAS according to groups (min. 50% improvement or a reduction of pain intensity by 3).

	Number (%) of Participants			*p* *
	With Herniated Disc and Nerve Contact	Without Herniated Disc and Nerve Contact	Total	
Improvement of baseline VAS vs. 1 month from ESI TF				
Improvement	20 (67)	21 (72)	41 (70)	0.63
No improvement	10 (33)	8 (28)	18 (31)	
In total	30 (100)	29 (100)	59 (100)	
Improvement of baseline VAS vs. 3 months from ESI TF				
Improvement	18 (60)	22 (76)	40 (68)	0.19
No improvement	12 (40)	7 (24)	19 (32)	
Total	30 (100)	29 (100)	59 (100)	

* Fisher’s exact test.

**Table 4:** ODI scores for all participants regarding measurements.

All Participants	Median (Interquartile Range)			*p* *
	Baseline	One Month from ESI TF	Three Months from ESI TF	
Pain intensity	4 (3–4)	3 (2–3)	3 (2–4)	<0.001 ^†^
Personal care	2 (2–3)	1 (1–3)	2 (1–2)	0.001 ^†^
Lifting	4 (3–5)	3 (2–4)	3 (2–4)	0.19
Walking	3 (2–4)	2 (1–3)	2 (1–3)	0.001 ^†^
Sitting	3 (3–4)	3 (3–4)	3 (3–4)	<0.001 ^†^
Standing	4 (3–4)	3 (2–4)	3 (2–4)	0.009 ^†^
Sleeping	2.5 (2–4)	2 (2–3)	2 (2–3)	0.001 ^†^
Sex life	3 (2–5)	2 (1–4)	2 (1–4)	0.03 ^‡^
Social life	3 (2–4)	3 (1–4)	3 (2–4)	0.05
Traveling	3 (2–4)	2 (2–3)	2 (2–4)	<0.001 ^†^
ODI in total (%)	64 (56–74)	54 (42–62)	54 (42–64)	<0.001 ^†^

* Friedman test (post hoc Conover test). ^†^ At the *p* < 0.05 level, values before ESI TF (baseline) were significantly higher than other measurements. ^‡^ At the *p* < 0.05 level, values before ESI TF (baseline) were significantly higher than 1 month after ESI TF.

**Table 5:** ODI values at three measurement points in groups with and without contact of disc herniation and nerve.

ODI	Median (Interquartile Range)			*p* *
	Baseline	One Month from ESI	Three Months from ESI	
With herniated disc and nerve contact				
Pain intensity	4 (3–4)	3 (2–4)	3 (2–4)	<0.001 ^†^
Personal care	2 (2–3)	1.5 (1–3)	2 (1–2)	0.10
Lifting	4 (3–5)	4 (3–4)	4 (2–4)	0.55
Walking	2 (2–4)	2 (1–3)	2 (2–3)	0.47
Sitting	3 (3–4)	3 (3–4)	3 (3–3)	0.10
Standing	3 (2–4)	3 (2–4)	3 (2–4)	0.78
Sleeping	2 (2–3)	2 (2–3)	2 (2–3)	0.20
Sex life	3 (2–5)	2 (1–4)	2 (1–5)	0.68
Social life	3 (2–4)	3 (2–4)	3 (2–4)	0.63
Traveling	3 (2–3)	2 (2–3)	2 (2–4)	0.21
Total ODI (%)	61 (52–74)	54 (44–68)	53 (42–64)	0.22
Without herniated disc and nerve contact				
Pain intensity	4 (3–4)	3 (1–3)	3 (2–3)	<0.001 ^†^
Personal care	2 (2–3)	1 (1–3)	1 (1–2)	0.005 ^†^
Lifting	4 (3–5)	3 (2–4)	3 (2–5)	0.18
Walking	3 (2–3)	2 (1–3)	2 (1–2)	<0.001 ^†^
Sitting	4 (3–4)	3 (2–4)	3 (3–4)	<0.001 ^†^
Standing	4 (3–5)	3 (2–4)	3 (2–4)	<0.001 ^†^
Sleeping	2 (2–4)	2 (2–2)	2 (2–2)	<0.001 ^†^
Sex life	3 (2–5)	2 (1–3)	2 (1–4)	0.01 ^‡^
Social life	4 (2–5)	3 (1–4)	3 (2–4)	0.007 ^†^
Traveling	3 (3–5)	2 (2–3)	2 (2–3)	<0.001 ^†^
Total ODI (%)	70 (60–74)	50 (36–60)	54 (42–64)	<0.001 ^†^

* Friedman test (post hoc Conover test). ^†^ At the *p* < 0.05 level, values before ESI TF (baseline) were significantly higher than other measurements. ^‡^ At the *p* < 0.05 level, values before ESI TF (baseline) were significantly higher than 1 month after ESI TF.

**Table 6:** Distribution of patients according to the ODI before ESI TF compared to 1 and 3 months after ESI TF among those with disc herniation and nerve contact.

With Herniated Disc and Nerve Contact	Number (%) of Participants According to the ODI before ESI TF					*p* *
	Moderate Disability	Severe Disability	Disabled Participants	Bedridden Participants	Total	
One month from ESI						
Minimum disability (OW 0–20%)	0	1	0	0	1 (3)	0.05
Moderate disability (OW 21–40%)	1	2	1	0	4 (13)	
Severe disability (OW 41–60%)	1	8	7	0	16 (53)	
Disabled participants (OW 61–80%)	0	2	5	0	7 (23)	
Bedridden participants (OW 81–100%)	0	0	1	1	2 (7)	
Total	2 (7)	13 (43)	14 (47)	1 (3)	30 (100)	
Three months from ESI						
Moderate disability (OW 21–40%)	1	4	2	0	7 (23)	0.25 †
Severe disability (OW 41–60%)	1	7	5	0	13 (43)	
Disabled participants (OW 61–80%)	0	2	5	1	8 (27)	
Bedridden participants (OW 81–100%)	0	0	2	0	2 (7)	
Total	2 (7)	13 (43)	14 (47)	1 (3)	30 (100)	

* Test of marginal homogeneity; † Wilcoxon test.

**Table 7:** Distribution of participants according to the ODI before ESI TF compared to 1 and 3 months after ESI TF among those without disc herniation and nerve contact.

Without Herniated Disc and Nerve Contact	Number (%) of Participants According to the ODI before ESI TF (Baseline)					*p* *
	Moderate Disability	Severe Disability	Disabled Participants	Bedridden Participants	Total	
One month from ESI						
Minimum disability (OW 0–20%)	0	2	6	0	8 (28)	<0.001
Moderate disability (OW 21–40%)	1	3	10	1	15 (52)	
Severe disability (OW 41–60%)	0	2	2	1	5 (17)	
Disabled participants (OW 61–80%)	0	0	0	1	1 (3)	
Total	1 (3)	7 (24)	18 (63)	3 (10)	29 (100)	
Three months from ESI						
Moderate disability (OW 21–40%)	0	2	5	0	7 (24)	0.001
Severe disability (OW 41–60%)	1	2	9	0	12 (41)	
Disabled participants (OW 61–80%)	0	3	4	3	10 (35)	
In total	1 (3)	7 (24)	18 (62)	3 (10)	18 (100)	

* Test of marginal homogeneity.

## Data Availability

The data presented in this study are available from the corresponding author upon request.
